# Prevalence of porcine circovirus type 3 in pigs in the southeastern Chinese province of Zhejiang

**DOI:** 10.1186/s12917-019-1977-7

**Published:** 2019-07-15

**Authors:** Shichao Geng, Hao Luo, Yajie Liu, Cong Chen, Weicheng Xu, Yunlu Chen, Xiaoliang Li, Weihuan Fang

**Affiliations:** Zhejiang University Institute of Preventive Veterinary Medicine and Zhejiang Provincial Key Laboratory of Preventive Veterinary Medicine, Hangzhou, 310058 Zhejiang China

**Keywords:** Porcine circovirus 3, Co-infection, Phylogenetic analysis, Sero-prevalence

## Abstract

**Background:**

Porcine circovirus type 3 (PCV3) was first reported in US in 2016. The virus was also identified later in China. Prevalence of PCV3 in Zhejiang province in southeastern China is not clear though it has been reported in many parts of China.

**Results:**

PCV3 infection and its co-infection with other swine viral pathogens in pig herds of Zhejiang province were retrospectively investigated by quantitative PCR (qPCR) and its sero-prevalence by indirect ELISA. PCV3 was found positive in 67.1% of the 283 clinical samples taken from 2014 to 2017 as shown by qPCR. Single infection with PCV3 accounted for only one-third of the samples, and majority were of co-infections, predominantly with PEDV (41.6%) but generally low with other swine viruses. Indirect ELISA using the PCV3 capsid protein as the coating antigen revealed an average sero-positive rate of 52.6% (40.8 to 60.8%) in 2345 serum samples from 2011 to 2017, with earliest yet high positive findings in samples taken in 2012. Of 203 serum samples, the qPCR method showed more positive findings than ELISA (81.3% vs 56.2%). With 89 serum samples negative by ELISA, vast majority (*n* = 81) were found positive by qPCR. There was negative correlation in levels of PCV3 DNA and anti-capsid antibody response. ORF2-based phylogenetic analysis revealed three major groups (PCV3a, PCV3b and PCV3c) of the 200 strains, 38 from this study and 162 reference strains from GenBank. Most of the strains from this study were clustered into PCV3c. Of the putative signature residues of the capsid protein (aa 24, 27, 77 and 150) relative to the three groups, only the PCV3a group strains showed a distinct pattern of residues VKSI (95% of the strains), while the other two groups did not have such a ‘signature’ pattern.

**Conclusions:**

Results from this study provided further evidence that the novel virus PCV3 was widely distributed in China and might have emerged in Zhejiang province before 2014, most probably back in 2012 when there was high PCV3 sero-prevalence. PCV3 might be viremic in pigs and could spread by fecal shedding.

**Electronic supplementary material:**

The online version of this article (10.1186/s12917-019-1977-7) contains supplementary material, which is available to authorized users.

## Background

Porcine circoviruses are small non-enveloped viruses with single-stranded circular DNA and belong to the genus Circovirus and the family Circoviridae [1]. Two types of PCVs (PCV1 [2] and PCV2 [3]) have been reported in pigs. PCV1 is non-pathogenic to pigs [4], while PCV2 is related to porcine circovirus-associated diseases (PCVAD) [5]. In 2016, a novel circovirus PCV3 was first detected in pigs in the US shown as porcine dermatitis and nephropathy syndrome (PDNS), reproductive failure, multi-systemic inflammation and cardiac pathology [6, 7]. The PCV3 genome is 2000 nucleotides in length with two major inversely arranged ORFs encoding replicase (Rep) and capsid (Cap) proteins [6, 7]. So far, the novel pathogen has been reported in major pig-producing countries including China, Poland, South Korea, Italy, Brazil, Germany, Thailand, Denmark, Spain, Sweden and Russia [8–17]. PCV3 was also identified in pigs with no clinical signs of infection or disease conditions [9–18] only [9, 18] after this sentence. Please delete ref numbers 10 to 17. Here we report the prevalence of PCV3 in pig herds in Zhejiang, a southeastern province of China, by molecular method and indirect ELISA and its co-infections with other major swine viral pathogens.

## Results

### Prevalence of PCV3 in Zhejiang province from 2014 to 2017

**Fig. 1:**
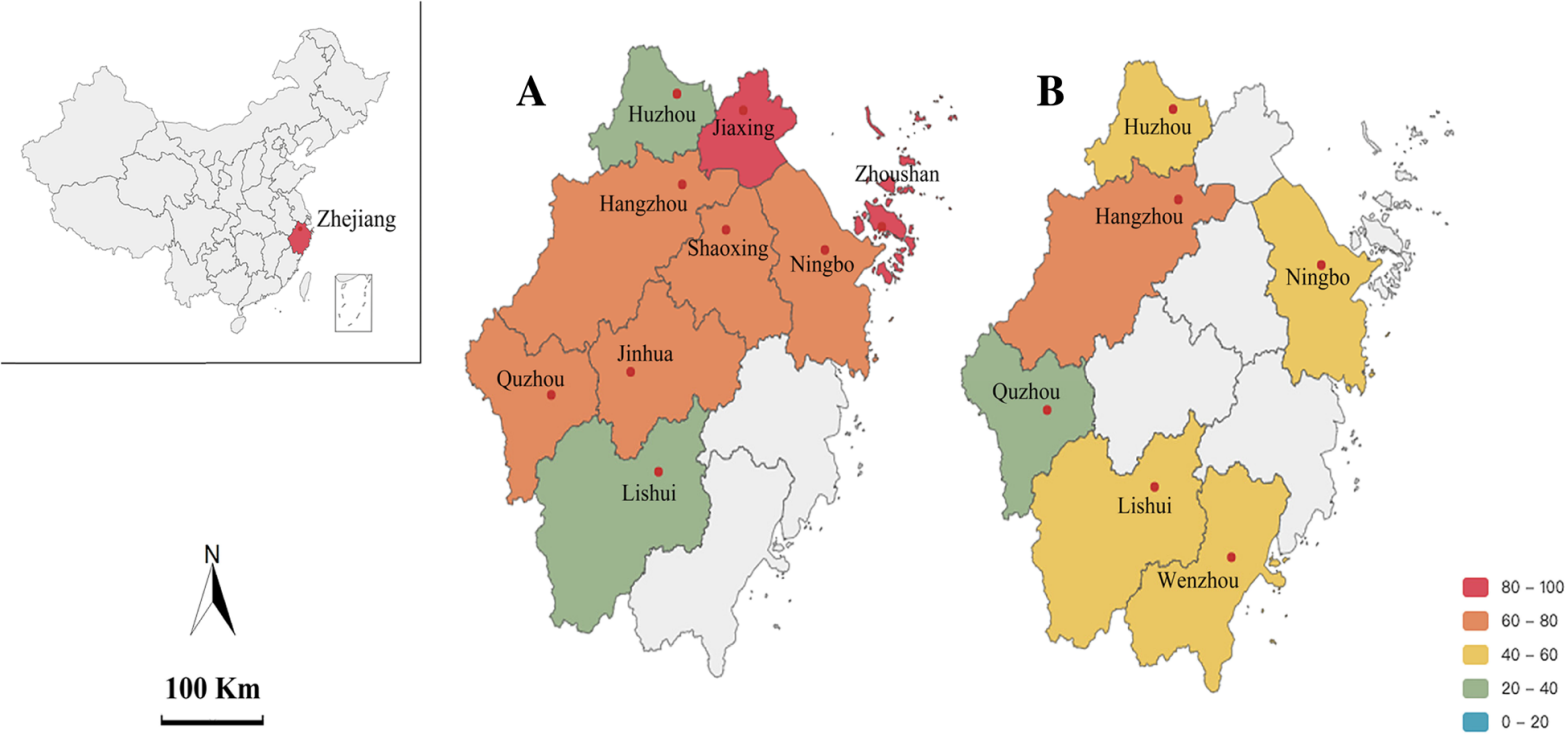
Geographical distribution of tissue samples (**a**, qPCR) and serum samples (**b**, indirect ELISA) with graded scales showing different levels of PCV3 infection. The insert shows the location of Zhejiang province (red) in the map of China. The frame diagram of the China map was from an open database of Pyecharts packages geo and map (http://pyecharts.herokuapp.com)

Of all 283 samples examined by qPCR, the overall prevalence of PCV3 was 67.1% (190/283) in Zhejiang. The PCV3-positive rate was 83.3, 72.3, 75.9 and 45.8% in 2014, 2015, 2016 and 2017, respectively (Table 1). Ct values of all the tested samples ranged from 21.42 to 37.31. Considering that PCV3 nucleic acid could be detected as early as 2014 at high percentage, the virus might have emerged in this part of China prior to 2014. The PCV3-positive rate varied from 37.5 to 85.7% among different sample sources (Fig. 1 and Additional file 1: Table S1). By sample types, the lung and lymph nodes showed higher rate than the small intestine and fecal samples (Table 2).

**Table 1:** Prevalence of PCV3 in 283 clinical samples from 2014 to 2017 as detected by qPCR

Year	No. of Samples	Range of Ct values	Positive samples(Ct < 30)	Positive rate (%)
2014	48	22.89–33.58	40	83.3
2015	94	22.71–33.48	68	72.3
2016	58	21.42–37.31	44	75.9
2017	83	24.28–34.10	38	45.8
Total	283		190	67.1

**Table 2:** Positive findings of PCV3 by sample types as determined by qPCR

Sample type	No. of samples	No. of positive samples	Positive rate (%)
Small intestine	216	149	69.0
Feces	38	17	44.7
Lymph node	21	17	81.0
lung	8	7	87.5
Total	283	190	67.1

### Co-infections of PCV3 with other swine viral pathogens

Co-infection of PCV2 with other pathogens has been reported [19–23]. We also examined if PCV3 was concurrent with major diarrheal viruses (porcine epidemic diarrhea virus [PEDV], porcine delta coronavirus [PDCoV] and group A rotavirus [GARV] as well as other viral pathogens (classical swine fever virus [CSFV], PCV2 and porcine reproductive and respiratory virus [PRRSV]). Of the 190 PCV3-positive samples, 36.3% was of single infection while 63.7% (121/190) showed mixed infections with other pathogens (Table 3). The positive rate of mixed infections of both PCV3 and PEDV was the highest (41.6%), followed by PCV3 and PCV2 (8.9%). Mixed infections of PCV3 with CSFV, PDCoV or PRRSV were low (from 1.1 to 2.6%). Besides, triple infection of PCV3 with two other pathogens was also noted, 5.8% with PCV2 and PEDV, and 1.6% with PEDV and PDCoV.

**Table 3:**
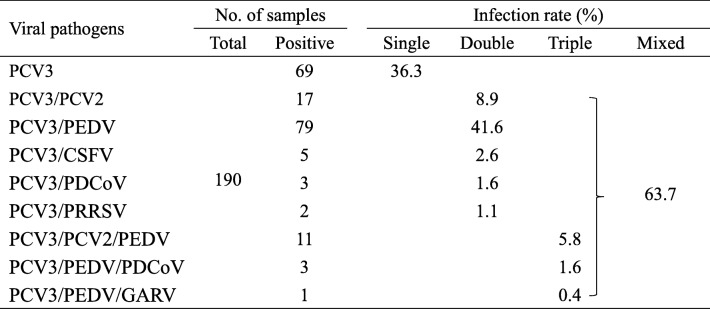
Co-infection of PCV3 with other swine viral pathogens

### Phylogenetic analysis of PCV3 ORF2

**Fig. 2:**
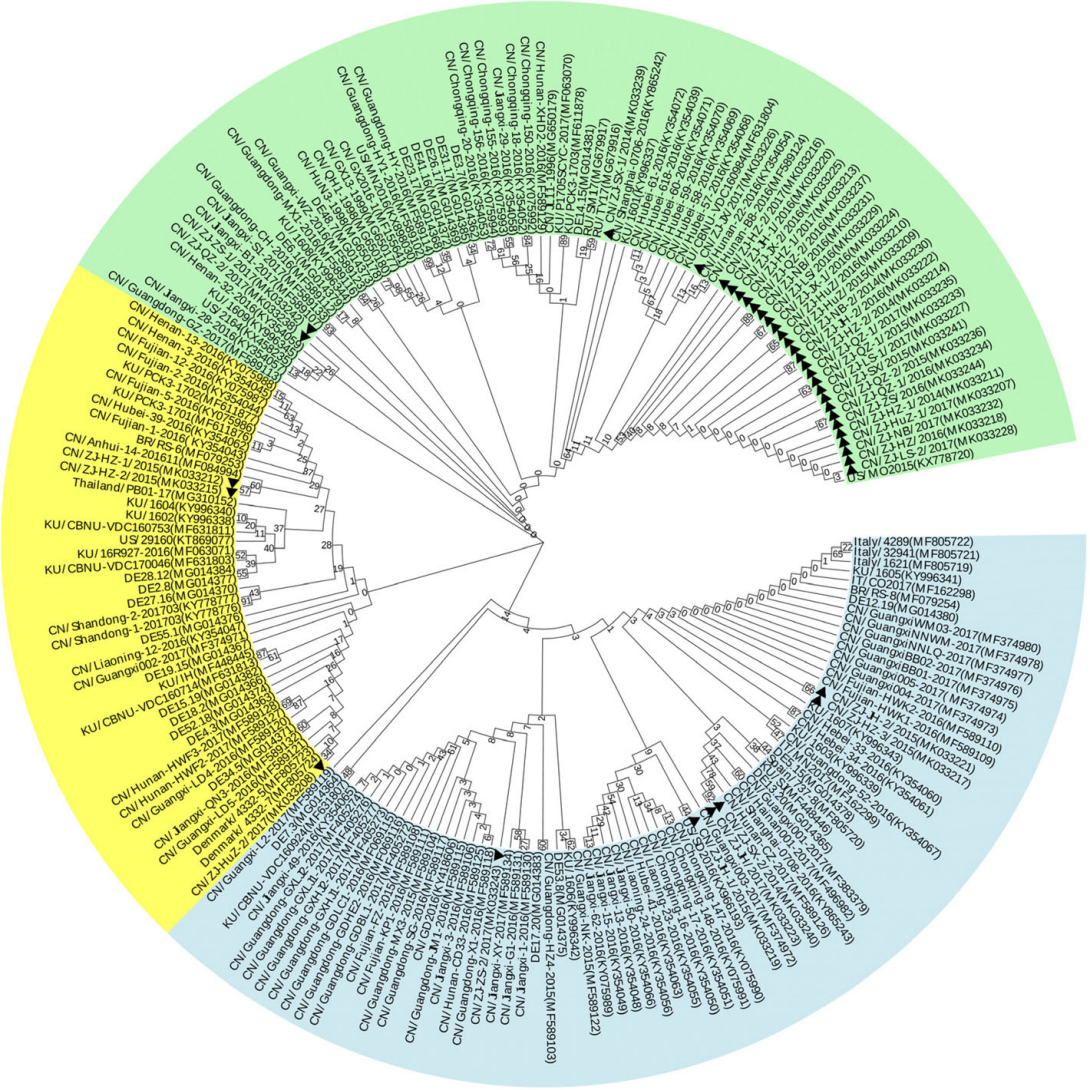
Phylogenetic analysis of the full-length ORF2 gene sequences of PCV3 based on 200 strains: 38 strains from this study indicated by black triangles and 162 strains downloaded from GenBank with accession number and strain information listed in Additional file 1: Table S1. All PCV3 strains could be divided into three groups, PCV3a (blue), PCV3b (yellow) and PCV3c (green). The tree was constructed by the neighbor-joining method in MEGA 7.0 software (p-distance model; 1000 bootstraps; only bootstrap scores above 50 are shown)

ORF2 from 38 PCV3-positive samples was sequenced. There was 97.1 to 100% nucleotide similarity among the ORF2 sequences, and their similarity with the USA isolate PCV3-US/MO2015 (KX778720.1) was from 97.7 to 99.8%. Two hundred ORF2 sequences of different countries (38 from this study and 162 downloaded from GenBank) were subjected to phylogenetic analysis. Fig. 2 shows that the PCV3 sequences could be divided into three major groups (PCV3a, PCV3b and PCV3c). The sequences from Zhejiang province were distributed in all three groups, while most of them were present in the group PCV3c and clustered into a small branch together with the USA isolate PCV3-US/MO2015.

Some studies reported the patterns of key amino acid residues 24, 27, 77 and 150 on PCV3 ORF2 [13, 24, 25]. We analyzed these four residues by extending to the 200 isolates of the three groups (Fig. 2). Of the 10 combinations, vast majority of residues at 24, 27,77 and 150 were VKSI for isolates of the PCV3a group (95%, 73/77). Most of the PCV3c group isolates were of ARSI (78%, 62/79). PCV3b group isolates were either ARSI (41%, 18/44) or ARTL (57%, 25/44) (Table 4).

**Table 4:** Possible relationship between PCV3 groups and key amino acid residues of the ORF2 (capsid) protein

Virus Group	No. of Sequences	Amino acid residues 24, 27, 77 and 150									
		ARSI	ARSL	AKSI	AKSF	VRSI	VKSI	VKNI	LKSI	ARTI	ARTL
PCV3a	77					2	73	1	1		
PCV3b	44	18								1	25
PCV3c	79	62	2	5	2	1	7				
Total	200	80	2	5	2	3	80	1	1	1	25

The putative ‘signature’ residues of the ORF2 protein (aa 24, 27, 77 and 150) relative to the PCV3 groups (PCV3a, PCV3b and PCV3c) were analyzed based on 200 strains, 38 obtained in this study and 162 downloaded from NCBI GenBank

### Sero-prevalence of PCV3 in pig herds in Zhejiang province

To understand the prevalence of PCV3 at the serum antibody level in pig populations in Zhejiang province, a retrospective serological survey was performed. A total of 2345 pig serum samples collected from 2011 to 2017 was analyzed by indirect ELISA. The PCV3 capsid protein was of good purity (Additional file 1: Figure S1), and did not cross-react with PCV2 positive sera (OD_450nm_ at 0.18 ± 0.01, Additional file 1: Table S2). With the OD_450nm_ cutoff value at 0.53 for differentiating PCV3-positive and -negative serum samples, the overall sero-prevalence was 52.6% (1234/2345), ranging from 46.8 to 60.8% for different years (Table 5). PCV3 antibody was detected from clinical samples as early as 2012 with the positive rate of 60.8% (535/880). The PCV3-positive rate ranged from 34.2 to 60.9% in different places of Zhejiang province (Fig. 1 and Additional file 1: Table S3).

**Table 5:** Sero-prevalence of PCV3 infection in pig serum samples from 2011 to 2017 as determined by indirect ELISA

Year	No. of samples	No. of positive samples	Positive rate (%)
2011	28	0	0
2012	880	535	60.8
2013	46	27	58.7
2016	663	310	46.8
2017	728	362	49.7
Total	2345	1234	52.6

### Comparison of PCV3 detection by qPCR and indirect ELISA in pig serum samples

**Fig. 3:**
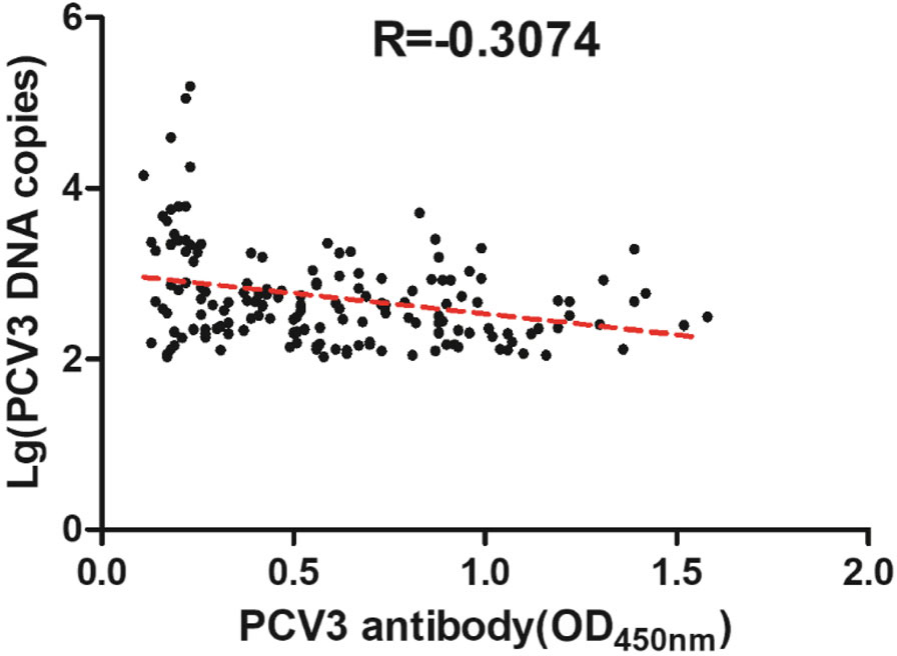
Correlation analysis between the levels of PCV3 DNA (lg DNA copies) and anti-PCV3-capsid antibody response (shown as OD_450nm_) in 165 pig serum samples. Pearson correlation coefficient (R) is −0.3074, *P* < 0.0001

Of the 203 serum samples randomly selected for comparison of qPCR and ELISA data, qPCR showed more positive samples than ELISA (81.3% vs 56.2%)(Table 6). With 89 serum samples negative by ELISA, vast majority (*n* = 81) were found positive by qPCR. These results indicate that these pigs might be under viremic conditions. Also there were samples positive by ELISA (*n* = 114), but negative by qPCR (*n* = 30, 26% [30/114]). A total of 165 pig serum samples positive by qPCR were further used for correlation analysis with indirect ELISA. The OD_450nm_ values of these samples were from 0.11 to 1.72, and the PCV3 DNA copies varied from 10^2.03^ to 10^5.19^. Fig. 3 reveals that the PCV3 DNA level was negatively correlated with the antibody response in pig serum samples (the Pearson correlation coefficient R at − 0.3074, *P* < 0.0001).

**Table 6:** Comparison between indirect ELISA and qPCR for detection of PCV3 in serum samples

		qPCR		Total (%)
		Positive No.	Negative No.	
ELISA	Positive No.	84	30	114 (56.2)
	Negative No.	81	8	89 (43.8)
	Total (%)	165 (81.3)	38 (18.7)	203

## Discussion

PCV3 has been reported in major pig-producing countries since the first report in the US in 2016 [6–17]. The virus was reported in China in samples taken in 2015 [8] with its possible presence traced back as early as 1996 as revealed in another study [25]. By qPCR, we found that PCV3 was present in two-thirds of the clinical samples including archived specimen back from 2014. Indirect ELISA revealed that the anti-PCV3-capsid antibody was found in nearly half of the archived serum samples from 2011. These findings suggest that PCV3 infection in pig farms in Zhejiang was significant both at the viral nucleic acid and serum antibody levels. With relatively stringent cutoff values of Ct (30 Ct by qPCR) and OD_450nm_ (0.53 by ELISA, refer to discussion below) positive for PCV3, we could infer that PCV3 might have emerged in this part of China prior to 2014, most probably back in 2012 when there was high PCV3 sero-prevalence and some of the archived serum samples were tested positive for PCV3 nucleic acids (21.6%, 11/51).

The PCV3-positive rates varied among reports by Chinese researchers. One study reported that 34.7% of the samples collected from 11 provinces were PCV3-positive [8]. In another report, the PCV3-positive rate was 59.4% [18]. Fu et al. (2018) reported that PCV3 was positive in 26.7% of their samples [24]. By sero-prevalence, Deng et al. (2018) reported that the PCV3 positive rate increased from 22.3% in 2015 to 51.8% in 2017 [26]. A recent US report revealed that 56.6% of their serum samples were positive [7]. Variations of the ELISA method used, particularly the purity of the coating antigen could have significant impact on determination of the cutoff values to differentiate positive samples from negative ones. Deng et al. (2018) set the cutoff value at 0.31 with average absorbance of 0.29 from 20 serum samples from cesarean-derived and colostrum-deprived pigs used as negative controls [26]. Palinski et al. reported the cutoff value at 0.87 (with mean OD at 0.49 of 18 serum samples from PCV3-negative SPF pigs [7]. In our study, a stringent cutoff value of 0.53 was set from the OD_450nm_ values of serum samples from 40 individual clinically healthy pigs. The capsid protein we used was of high purity and did not cross-react with PCV2 sera.

Co-infection of PCV3 with other pig viral pathogens has been reported [6–8, 24, 27–30]. Co-infection of PCV3 and PCV2 varied from 15.8 to 39.4% [8, 18, 24]. PRRSV was found in 25% of the PCV3-positive stillbirth samples [24] and 20.7% of the pigs with severe respiratory disease [27]. Co-infection of PCV3 with TTSuV1 and TTSuV2 was also reported at high percentage from 50 to 83.3% [29]. Our study reveals that single infection with PCV3 was found only in one-third of the samples and majority were of co-infections with PEDV, PCV2, CSFV, GARV, PDCoV or PRRSV. However, double co-infection rates varied greatly, the highest with PEDV (41.6%). PCV3 co-infections with other swine viruses were generally low. Triple infections of PCV3 with PCV2 and PEDV as well as with PEDV and PDCoV were also seen, but at low percentage.

Although there is high identity among the PCV3 strains of this study or among the Chinese isolates or between isolates from China and those from other countries at the nucleotide or amino acid levels. Genetic diversity does exist. Fux et al. (2018) reported that the PCV3 strains could be divided into two main groups based on the complete genome of 45 isolates [13]. Phylogenetic analysis of the ORF2 genes also showed that the viral strains could be grouped into 3 clades [24]. Here we show that there are three major groups (PCV3a, PCV3b and PCV3c) based on the ORF2 sequences of 200 strains. While most of the strains in this study were clustered into PCV3c, there were also strains in the other two groups. Regarding putative signature residues of the ORF2 protein, there did exist some patterns at residues 24, 27, 77 and 150 relative to the three groups of strains [13]. However, only the PCV3a group strains showed a distinct pattern of residues VKSI (95% of the strains).

Because most of the clinical and serum samples in this investigation were archived ones, it is impossible to examine the relationship between qPCR and ELISA results by sample match. To investigate the degree of agreement between the two methods or the correlation between the levels of antibody response and viral DNA copies, a total of 203 pig serum samples were randomly selected for indirect ELISA and qPCR. It is clear that qPCR had more positive findings than ELISA (81.3% vs 56.2%, Table 6). If sero-conversion is considered as a reference, the agreement was about 73% (84/114). The discrepancy resulted mainly from the negative samples by ELISA that were found positive by qPCR (91%, 81/89), suggesting that these pigs might be viremic before sero-conversion. It seems that the higher the antibody level, the lower the amount of viral nucleic acid (Fig. 3). This might suggest that the virus was eliminated by the antibody response, thus explaining the negative findings by qPCR in 26% of the ELISA positive samples (30/114).

## Conclusions

The present study further extends the evidence that the novel virus PCV3 has been widely distributed in Zhejiang, China several years before the first report in the US in 2016. Co-infection of PCV3 with PEDV in Zhejiang was more pronounced than with other swine viral pathogens. PCV3 might be viremic in pigs and could spread by fecal shedding.

## Methods

### Porcine clinical samples, serum samples and pre-treatment

A total of 283 clinical samples were collected from diseased pigs on farms in Zhejiang provinces, China between 2014 and 2017 (Fig. 1). The samples were initially used for detection of porcine epidemic diarrhea virus. The sample types included small intestines (216), feces (38), lymph node (21) and lung (8). The samples were homogenized in phosphate buffered saline (PBS, 10 mM, pH 7.4) and subjected to 2 cycles of freezing and thawing, followed by vortexing for 5 min and centrifugation at 135,000 x g (Eppendorf, Germany) for 10 min at 4 °C. The supernatants were collected and stored at − 80 °C refrigerator for DNA (DNA viruses) and RNA (RNA viruses) extraction. The archived serum samples (*n* = 2345) collected in Zhejiang province from 2011 to 2017 and kept frozen at − 20 °C were initially used for detecting PEDV antibodies and then tested for anti-PCV3 antibodies. A written consent from farm owners was obtained for porcine clinical samples and serum samples used in this study. For sampling of organs from clinically ill pigs, anesthetic agents pentabarbitol sodium (30 mg/kg) and thiopental sodium (20 mg/kg) were used in combination by intravenous infusion for euthanasia.

### Detection of PCV3 DNA and other viral pathogens by quantitative PCR

Viral DNA and RNA were extracted from 283 clinical samples using the FavorPre Viral Nucleic Acid Extraction Kits (Favorgen, Austria) following the manufacture’s instruction. PCV3 DNA was detected by quantitative PCR (qPCR) with the THUNDERBIRD probe qPCR Mix (Toyobo, Japan) as previously described [7]. This qPCR protocol had the limit of detection at 10^2^ copies (Ct at 33–34) using a serial dilution of a recombinant plasmid containing PCV3 genome using pUC57 as the backbone. We consider Ct values less than 30 as positive for PCV3.

For PCV3-positive samples, qPCR (reverse transcription qPCR for RNA viruses) was also used to examine possible co-infections of PCV3 with other major swine viral pathogens using previous protocols (classical swine fever virus [31], group A rotavirus [32], PCV2 [33], porcine delta coronavirus [34], porcine epidemic diarrhea virus [35] and porcine reproductive and respiratory virus [36]).

### PCR amplification, sequencing and phylogenetic analysis

The full-length ORF2 of 38 PCV3-positive samples were amplified as previously described [24] using the PrimeSTAR Max DNA Polymerase (Takara, China). The PCR products were separated by electrophoresis on 1.0% agarose gels, purified with the Gel Extraction Kit (Omega, China) and cloned into the pEASY-Blunt Zero Cloning Kit (TransGen Biotech, China) for sequencing (Biosune Co. Ltd., Shanghai, China). A total of 162 PCV3 sequences from Brazil, other parts of China, Denmark, Germany, Italy, Russia, South Korea, Spain, Thailand and USA were downloaded from NCBI GenBank (Additional file 1: Table S4). The phylogenetic tree was constructed by the neighbor-joining method in MEGA 7.0 software (p-distance model, 1000 bootstraps).

### Prokaryotic expression and identification of PCV3 capsid protein

The ORF2 gene fragment (without the putative nuclear localization signal) was amplified as previously described [7] and the PCR product was cloned into the pET30a plasmid with the ClonExpress II One Step Cloning Kit (Vazyme, China). Then the recombinant plasmid pET30a-cap was transformed into *E. coli* BL21(DE3) cells for expression of a truncated capsid protein as previously described [37]. The recombinant protein was then purified using the Ni^2+^-NTA agarose column and used as the coating antigen for indirect ELISA. The purified cap protein was subjected to SDS-PAGE and Western blotting with the rabbit anti-PCV3-cap serum and anti-6 × His monoclonal antibody (Sangon Biotech, China) (Additional file 1: Figure S1).

### Indirect ELISA for detection of PCV3 antibodies

To detect the PCV3 antibodies in serum samples, indirect ELISA was employed as previously described [26] using overnight coating, at 4 °C, of the purified capsid protein coating (2 μg/well) on EIA/RIA high binding plates (Corning, USA). The plates were then blocked with 0.1% casein for 2 h at 37 °C. Serum samples were diluted to 1:200. Peroxidase-conjugated goat anti-swine IgG (Proteinteck, China) was used as secondary antibody at 1:10000 dilution. The coloring reagent was tetramethyl benzidine (TMB) kit (Jingqi Biological, China). The plates were measured at OD_450nm_ on the microplate reader (Epoch, Biotek, USA). The cutoff value differentiating positive and negative serum samples was set at 0.53 OD_450nm_, that is, three units of standard deviation above the mean of the negative control sera [7, 26] (average OD_450nm_ = 0.284 tested on 40 serum samples from clinically healthy pigs with standard deviation of 0.083). The coating antigen PCV3 cap was also tested on PCV2-positive serum samples to examine the specificity of closely related virus infection using the established ELISA protocol.

### Correlation analysis between the levels of PCV3 DNA and anti-capsid antibody in pig serum

To determine whether there is a correlation between the levels of PCV3 DNA and anti-capsid antibody response, a total of 203 pig serum samples were randomly selected to extract DNA for qPCR detection of PCV3 nucleic acid as described above. The results were compared with the indirect ELISA data. With 165 serum samples tested positive by qPCR, PCV3 DNA copy numbers in the serum samples were calculated according to the standard curve established in this study: Ct = − 3.4304 × (lg[PCV3 DNA copies]) + 41.008, *R*^2^ = 0.9991). The viral DNA copies were log-transformed for correlation analysis with the ELISA data using GraphPad Prism 5 software.

## Additional file

Additional file 1: **Table S1.** Geographical distribution of 283 clinical samples from Zhejiang province of China and PCV3 prevalence as detected by qPCR. **Figure S1.** Identification of PCV3 capsid protein in *E. coli* by SDS-PAGE (A) and Western blotting using rabbit anti-PCV3-capsid serum (B) and mouse anti-His monoclonal antibody (C). M: protein marker in KDa. Cap: PCV3-capsid protein purified by Ni^2+^-NTA affinity column. **Table S2.** Evaluation of the in-house specificity of the indirect ELISA using PCV3 capsid protein as the coating antigen for differential detection of PCV3 and PCV2 antibodies in 30 PCV2-positive and 30 PCV3 positive serum samples. **Table S3.** Geographical distribution of pig serum samples from Zhejiang province of China and sero-prevalence of PCV3 infection as determined by indirect ELISA. **Table S4.** PCV3 strains used for sequence alignment and phylogenetic analysis. (DOCX 2126 kb)

## Data Availability

The ORF2 sequences of 38 PCV3 strains obtained in this study have been deposited and available at NCBI GenBank with the accession number MK033207-MK033244. The 162 reference strains downloaded from NCBI GenBank are listed in the Additional file 1: Table S4.

## Abbreviations

Cap: Capsid
CSFV: Classical swine fever virus
Ct: Cycle threshold
ELISA: Enzyme linked immunosorbent assay
GARV: Group A rotavirus
MEGA: Molecular Evolutionary Genetics Analysis
ORF: Open reading frame
PBS: Phosphate buffered saline
PCV1: Porcine circovirus type 1
PCV2: Porcine circovirus type 2
PCV3: Porcine circovirus type 3
PCVAD: Porcine circovirus-associated diseases
PDCoV: Porcine delta coronavirus
PDNS: Porcine dermatitis and nephropathy syndrome
PEDV: Porcine epidemic diarrhea virus
PRRSV: Porcine reproductive and respiratory virus
qPCR: Quantitative real-time PCR
Rep: Replicase
TMB: Tetramethyl benzidine
TTSuV: Torque teno sus virus
