# Supplementary material for: Prevalence of porcine circovirus type 3 in pigs in the southeastern Chinese province of Zhejiang
Source: BMC Vet Res. 2019 Jul 15;15:244. doi: 10.1186/s12917-019-1977-7 (PMC6631677; doi:10.1186/s12917-019-1977-7)
Supplement: Supplementary file 1 — Table S1. Geographical distribution of 283 clinical samples from Zhejiang province of China and PCV3 prevalence as detected by qPCR. Figure S1. Identification of PCV3 capsid protein in E. coli by SDS-PAGE (A) and Western blotting using rabbit anti-PCV3-capsid serum (B) and mouse anti-His monoclonal antibody (C). M: protein marker in KDa. Cap: PCV3-capsid protein purified by Ni2+-NTA affinity column. Table S2. Evaluation of the in-house specificity of the indirect ELISA using PCV3 capsid protein as the coating antigen for differential detection of PCV3 and PCV2 antibodies in 30 PCV2-positive and 30 PCV3 positive serum samples. Table S3. Geographical distribution of pig serum samples from Zhejiang province of China and sero-prevalence of PCV3 infection as determined by indirect ELISA. Table S4. PCV3 strains used for sequence alignment and phylogenetic analysis. (DOCX 2126 kb) [file 12917_2019_1977_MOESM1_ESM.docx]

**Supplemental Table S1.** Geographical distribution of 283 clinical samples from Zhejiang province of China and PCV3 prevalence as detected by qPCR

| Areas | No. of samples | No. of positive samples | Positive rate (%) |
| --- | --- | --- | --- |
| Hangzhou | 129 | 81 | 62.8 |
| Huzhou | 8 | 3 | 37.5 |
| Jinhua | 50 | 40 | 80.0 |
| Jiaxing | 12 | 10 | 83.3 |
| Lishui | 8 | 3 | 37.5 |
| Ningbo | 19 | 12 | 63.2 |
| Quzhou | 29 | 21 | 72.4 |
| Shaoxing | 21 | 14 | 66.7 |
| Zhoushan | 7 | 6 | 85.7 |
|  |  |  |  |
| Total | 283 | 190 | 67.1 |

70


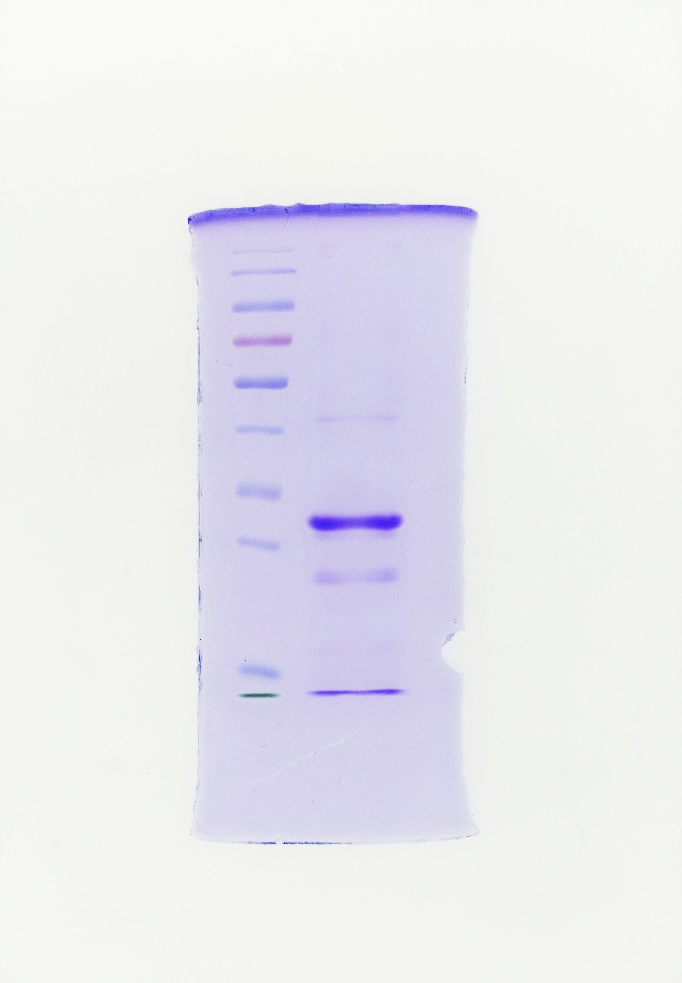


Cap

M

55

40

35

25

**A**


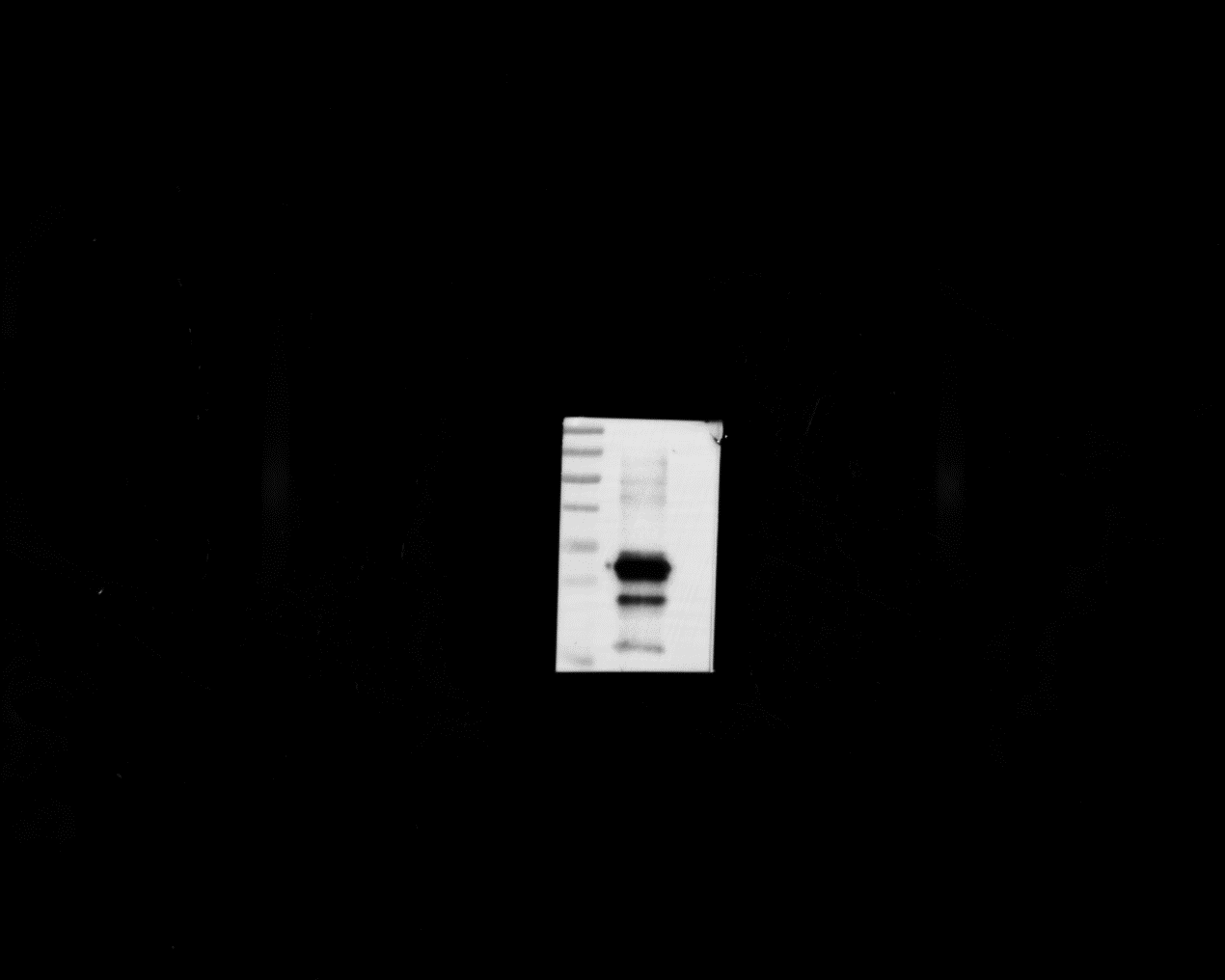


70

Cap

M

55

40

35

25

**B**


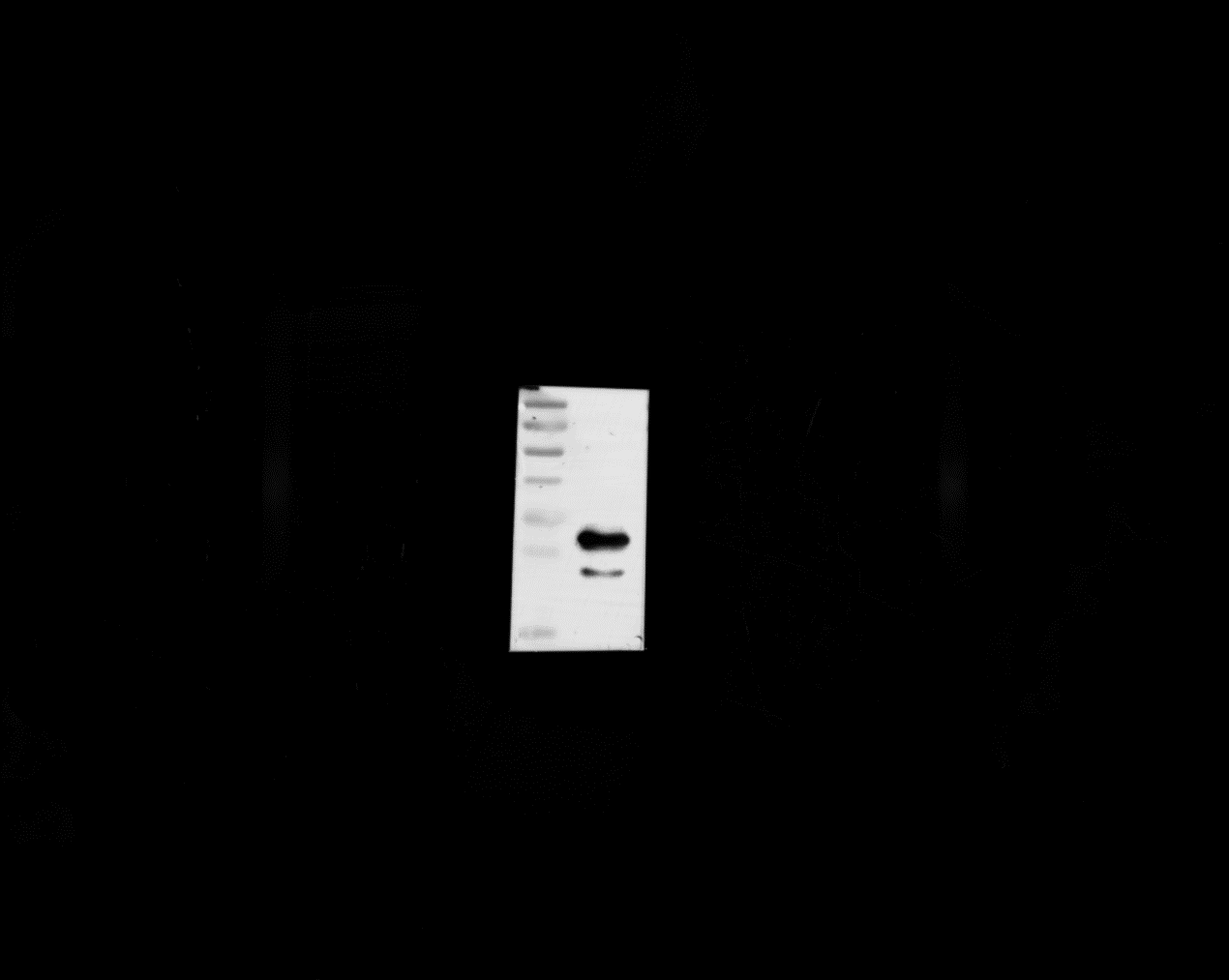


70

Cap

M

55

40

35

25

**C**

**Supplemental Figure S1**: Identification of PCV3 capsid protein in *E. coli* by SDS-PAGE (A) and Western blotting using rabbit anti-PCV3-capsid serum (B) and mouse anti-His monoclonal antibody (C).

M: protein marker in KDa. Cap: PCV3-capsid protein purified by Ni^2+^-NTA affinity column.

**Supplemental Table S2**. Evaluation of the in-house specificity of the indirect ELISA using PCV3 capsid protein as the coating antigen for differential detection of PCV3 and PCV2 antibodies in 30 PCV2-positive and 30 PCV3 positive serum samples.

| Serum samples | OD_450nm_ | | |
| --- | --- | --- | --- |
|  | PCV2-Cap |  | PCV3 Cap |
| PCV2-positive (n=30) | 1.43 ± 0.05 |  | 0.18 ± 0.01 |
| PCV3-positive (n=30) | 0.203 ± 0.01 |  | 0.75 ± 0.03 |

**Supplemental Table S3.** Geographical distribution of pig serum samples from Zhejiang province of China and sero-prevalence of PCV3 infection as determined by indirect ELISA.

| Areas | No. of samples | No. of positive samples | Positive rate (%) |
| --- | --- | --- | --- |
| Hangzhou | 872 | 531 | 60.9 |
| Huzhou | 111 | 56 | 50.5 |
| Lishui | 49 | 26 | 53.1 |
| Ningbo | 1036 | 515 | 49.7 |
| Quzhou | 222 | 76 | 34.2 |
| Wenzhou | 55 | 30 | 54.5 |
|  |  |  |  |
| Total | 2345 | 1234 | 52.6 |

**Supplemental Table S4**: PCV3 strains used for sequence alignment and phylogenetic analysis.

| **No.** | **Strain Name** | **GenBank**  **Accession No.** | **Country** | **Collection Date** |
| --- | --- | --- | --- | --- |
| 1 | PCV3-BR/RS/6 | MF079253 | Brazil | 2016 |
| 2 | PCV3-BR/RS/8 | MF079254 | Brazil | 2016 |
| 3 | PCV3/CN/Anhui-14/201611 | MF084994 | China | 2016 |
| 4 | PCV3/CN/Chongqing-147/2016 | KY075990 | China | 2016 |
| 5 | PCV3/CN/Chongqing-148/2016 | KY075991 | China | 2016 |
| 6 | PCV3/CN/Chongqing-150/2016 | KY075992 | China | 2016 |
| 7 | PCV3/CN/Chongqing-155/2016 | KY075993 | China | 2016 |
| 8 | PCV3/CN/Chongqing-156/2016 | KY075994 | China | 2016 |
| 9 | PCV3/CN/Chongqing-16/2016 | KY354050 | China | 2016 |
| 10 | PCV3/CN/Chongqing-17/2016 | KY354051 | China | 2016 |
| 11 | PCV3/CN/Chongqing-18/2016 | KY354052 | China | 2016 |
| 12 | PCV3/CN/Chongqing-20/2016 | KY354053 | China | 2016 |
| 13 | PCV3/CN/Fujian-5/2016 | KY075986 | China | 2016 |
| 14 | PCV3/CN/Fujian-12/2016 | KY075987 | China | 2016 |
| 15 | PCV3/CN/Fujian-1/2016 | KY354043 | China | 2016 |
| 16 | PCV3/CN/Fujian-2/2016 | KY354044 | China | 2016 |
| 17 | PCV3/CN/Fujian-FZ/2015 | MF589108 | China | 2015 |
| 18 | PCV3/CN/Fujian-HWK1/2016 | MF589109 | China | 2016 |
| 19 | PCV3/CN/Fujian-HWK2/2016 | MF589110 | China | 2016 |
| 20 | PCV3/CN/Fujian-KP1/2016 | MF589111 | China | 2016 |
| 21 | PCV3/CN/Guangdong-52/2016 | KY354067 | China | 2016 |
| 22 | PCV3-China/GD2016 | KY418606 | China | 2016 |
| 23 | PCV3/CN/GDLC1/2016 | MF069115 | China | 2016 |
| 24 | PCV3/CN/GDHE2/2016 | MF069116 | China | 2016 |
| 25 | PCV3/CN/GDBL1/2017 | MF405272 | China | 2017 |
| 26 | PCV3/CN/Guangdong-HY1/2016 | MF589102 | China | 2016 |
| 27 | PCV3/CN/Guangdong-HZ4/2015 | MF589103 | China | 2015 |
| 28 | PCV3/CN/Guangdong-MX3/2015 | MF589104 | China | 2015 |
| 29 | PCV3/CN/Guangdong-CH/2016 | MF589112 | China | 2016 |
| 30 | PCV3/CN/Guangdong-GZ/2017 | MF589113 | China | 2017 |
| 31 | PCV3/CN/Guangdong-HY/2016 | MF589114 | China | 2016 |
| 32 | PCV3/CN/Guangdong-JM1/2016 | MF589115 | China | 2017 |
| 33 | PCV3/CN/Guangdong-MX1/2016 | MF589116 | China | 2016 |
| 34 | PCV3/CN/Guangdong-SG/2016 | MF589117 | China | 2016 |
| 35 | PCV3/CN/Guangdong-X1/2016 | MF589118 | China | 2016 |
| 36 | PCV3-China/GX2016-1 | MF155641 | China | 2016 |
| 37 | PCV3/CN/Guangxi002/2017 | MF374971 | China | 2017 |
| 38 | PCV3/CN/Guangxi003/2017 | MF374972 | China | 2017 |
| 39 | PCV3/CN/Guangxi004/2017 | MF374973 | China | 2017 |
| 40 | PCV3/CN/Guangxi005/2017 | MF374974 | China | 2017 |
| 41 | PCV3/CN/GuangxiBB01/2017 | MF374975 | China | 2017 |
| 42 | PCV3/CN/GuangxiBB02/2017 | MF374976 | China | 2017 |
| 43 | PCV3/CN/GuangxiNNLQ/2017 | MF374977 | China | 2017 |
| 44 | PCV3/CN/GuangxiNNWM/2017 | MF374978 | China | 2017 |
| 45 | PCV3/CN/GuangxiWM03/2017 | MF374980 | China | 2017 |
| 46 | PCV3/CN/Guangxi001/2017 | MF383379 | China | 2017 |
| 47 | PCV3/CN/GXHJ1/2017 | MF405273 | China | 2017 |
| 48 | PCV3/CN/GXLJ2/2017 | MF405274 | China | 2017 |
| 49 | PCV3/CN/GXLJ1/2017 | MF405276 | China | 2017 |
| 50 | PCV3/CN/GXHJ2/2017 | MF405277 | China | 2017 |
| 51 | PCV3/CN/Guangxi-L2/2017 | MF589119 | China | 2017 |
| 52 | PCV3/CN/Guangxi-LD4/2016 | MF589120 | China | 2016 |
| 53 | PCV3/CN/Guangxi-LD5/2016 | MF589121 | China | 2016 |
| 54 | PCV3/CN/Guangxi-NK/2015 | MF589122 | China | 2015 |
| 55 | PCV3/CN/Guangxi-WZ/2016 | MF589123 | China | 2016 |
| 56 | PCV3/CN/Hainan001/2017 | MF496982 | China | 2017 |
| 57 | PCV3/CN/Hebei-33/2016 | KY354060 | China | 2016 |
| 58 | PCV3/CN/Hebei-34/2016 | KY354061 | China | 2016 |
| 59 | PCV3/CN/Henan-13/2016 | KY075988 | China | 2016 |
| 60 | PCV3/CN/Henan-3/2016 | KY354045 | China | 2016 |
| 61 | PCV3/CN/Henan-32/2016 | KY354059 | China | 2016 |
| 62 | CN/Hubei-618/2016 | KY354039 | China | 2016 |
| 63 | PCV3/CN/Hubei-39/2016 | KY354062 | China | 2016 |
| 64 | PCV3/CN/Hubei-41/2016 | KY354063 | China | 2016 |
| 65 | PCV3/CN/Hubei-57/2016 | KY354068 | China | 2016 |
| 66 | PCV3/CN/Hubei-58/2016 | KY354069 | China | 2016 |
| 67 | PCV3/CN/Hubei-59/2016 | KY354070 | China | 2016 |
| 68 | PCV3/CN/Hubei-60/2016 | KY354071 | China | 2016 |
| 69 | PCV3/CN/Hubei-61/2016 | KY354072 | China | 2016 |
| 70 | PCV3/CN/Hunan-22/2016 | KY354054 | China | 2016 |
| 71 | PCV3/CN/Hunan-B8/2016 | MF589124 | China | 2016 |
| 72 | PCV3/CN/Hunan-CD33/2016 | MF589125 | China | 2016 |
| 73 | PCV3/CN/Hunan-CZ/2017 | MF589126 | China | 2017 |
| 74 | PCV3/CN/Hunan-HWF2/2017 | MF589127 | China | 2017 |
| 75 | PCV3/CN/Hunan-HWF3/2017 | MF589128 | China | 2017 |
| 76 | PCV3/CN/Hunan-XHD2/2016 | MF589129 | China | 2016 |
| 77 | PCV3/CN/Jiangxi-62/2016 | KY075989 | China | 2016 |
| 78 | PCV3/CN/Jiangxi-13/2016 | KY354048 | China | 2016 |
| 79 | PCV3/CN/Jiangxi-15/2016 | KY354049 | China | 2016 |
| 80 | PCV3/CN/Jiangxi-28/2016 | KY354057 | China | 2016 |
| 81 | PCV3/CN/Jiangxi-29/2016 | KY354058 | China | 2016 |
| 82 | PCV3/CN/Jiangxi-49/2016 | KY354065 | China | 2016 |
| 83 | PCV3/CN/Jiangxi-50/2016 | KY354066 | China | 2016 |
| 84 | PCV3/CN/Jiangxi-3/2016 | MF589106 | China | 2016 |
| 85 | PCV3/CN/Jiangxi-B1/2017 | MF589107 | China | 2017 |
| 86 | PCV3/CN/Jiangxi-1/2016 | MF589130 | China | 2016 |
| 87 | PCV3/CN/Jiangxi-G1/2016 | MF589131 | China | 2016 |
| 88 | PCV3/CN/Jiangxi-QN3/2016 | MF589132 | China | 2016 |
| 89 | PCV3/CN/Jiangxi-S1/2017 | MF589133 | China | 2017 |
| 90 | PCV3/CN/Jiangxi-XY/2017 | MF589134 | China | 2017 |
| 91 | PCV3/CN/Liaoning-12/2016 | KY354047 | China | 2016 |
| 92 | PCV3/CN/Liaoning-23/2016 | KY354055 | China | 2016 |
| 93 | PCV3/CN/Liaoning-24/2016 | KY354056 | China | 2016 |
| 94 | PCV3/CN/Shandong-1/201703 | KY778776 | China | 2017 |
| 95 | PCV3/CN/Shandong-2/201703 | KY778777 | China | 2017 |
| 96 | CHN_Shanghai_0706_2016 | KY865242 | China | 2016 |
| 97 | CHN_Shanghai_0708_2016 | KY865243 | China | 2016 |
| 98 | Porcine circovirus 3 isolate JL11-1996 | MG650179 | China | 1996 |
| 99 | Porcine circovirus 3 isolate HuN3-1998 | MG650178 | China | 1998 |
| 100 | Porcine circovirus 3 isolate GX13-1999 | MG650177 | China | 1999 |
| 101 | Porcine circovirus 3 isolate QH1-1998 | MG650180 | China | 1998 |
| 102 | Porcine circovirus 3 isolate 4332-7_Denmark_2017 | MF805724 | Denmark | 2017 |
| 103 | Porcine circovirus 3 isolate 4332-5_Denmark_2017 | MF805723 | Denmark | 2017 |
| 104 | DE3.7 | MG014362 | Germany | 2015 |
| 105 | DE4.3 | MG014363 | Germany | 2015 |
| 106 | DE7.3 | MG014364 | Germany | 2015 |
| 107 | DE13.20 | MG014365 | Germany | 2015 |
| 108 | DE18.2 | MG014366 | Germany | 2015 |
| 109 | DE19.15 | MG014367 | Germany | 2015 |
| 110 | DE23.17 | MG014368 | Germany | 2015 |
| 111 | DE26.17 | MG014369 | Germany | 2015 |
| 112 | DE27.16 | MG014370 | Germany | 2015 |
| 113 | DE34.5 | MG014371 | Germany | 2015 |
| 114 | DE41.16 | MG014372 | Germany | 2015 |
| 115 | DE48.7 | MG014373 | Germany | 2015 |
| 116 | DE52.18 | MG014374 | Germany | 2015 |
| 117 | DE53.8 | MG014375 | Germany | 2015 |
| 118 | DE55.1 | MG014376 | Germany | 2015 |
| 119 | DE2.8 | MG014377 | Germany | 2015 |
| 120 | DE5.15 | MG014378 | Germany | 2015 |
| 121 | DE6.1 | MG014379 | Germany | 2015 |
| 122 | DE12.19 | MG014380 | Germany | 2015 |
| 123 | DE14.15 | MG014381 | Germany | 2015 |
| 124 | DE15.19 | MG014382 | Germany | 2015 |
| 125 | DE17.20 | MG014383 | Germany | 2015 |
| 126 | DE28.12 | MG014384 | Germany | 2015 |
| 127 | DE31.17 | MG014385 | Germany | 2015 |
| 128 | Porcine circovirus 3 strain PCV3-RU/SM17 | MG679917 | Russia | 2017 |
| 129 | Porcine circovirus 3 strain PCV3-RU/TY17 | MG679916 | Russia | 2017 |
| 130 | PCV3/KU-1601 | KY996337 | South Korea | 2016 |
| 131 | PCV3/KU-1602 | KY996338 | South Korea | 2016 |
| 132 | PCV3/KU-1603 | KY996339 | South Korea | 2016 |
| 133 | PCV3/KU-1604 | KY996340 | South Korea | 2016 |
| 134 | PCV3/KU-1605 | KY996341 | South Korea | 2016 |
| 135 | PCV3/KU-1606 | KY996342 | South Korea | 2016 |
| 136 | PCV3/KU-1607 | KY996343 | South Korea | 2016 |
| 137 | PCV3/KU-1608 | KY996344 | South Korea | 2016 |
| 138 | PCV3/KU-1609 | KY996345 | South Korea | 2016 |
| 139 | Porcine circovirus 3 strain 16R927/2016 | MF063071 | South Korea | 2016 |
| 140 | Porcine circovirus 3 strain P1705SCYC/2017 | MF063070 | South Korea | 2017 |
| 141 | Porcine circovirus 3 isolate CBNU-VDC160714 | MF631813 | South Korea | 2017 |
| 142 | Porcine circovirus 3 isolate CBNU-VDC160753 | MF631811 | South Korea | 2017 |
| 143 | Porcine circovirus 3 isolate CBNU-VDC160924 | MF631807 | South Korea | 2017 |
| 144 | Porcine circovirus 3 isolate CBNU-VDC160984 | MF631804 | South Korea | 2017 |
| 145 | Porcine circovirus 3 isolate CBNU-VDC170046 | MF631803 | South Korea | 2017 |
| 146 | Porcine circovirus 3 isolate SJ | MF448446 | South Korea | 2017 |
| 147 | Porcine circovirus 3 isolate IH | MF448445 | South Korea | 2017 |
| 148 | Porcine circovirus 3 strain PCK3-1703 | MF611878 | South Korea | 2017 |
| 149 | Porcine circovirus 3 strain PCK3-1702 | MF611877 | South Korea | 2017 |
| 150 | Porcine circovirus 3 strain PCK3-1701 | MF611876 | South Korea | 2017 |
| 151 | Porcine circovirus 3 isolate 737-8 | MF805720 | Spain | 2017 |
| 152 | PCV3-IT/CO2017 | MF162298 | Ttaly | 2017 |
| 153 | PCV3-IT/MN2017 | MF162299 | Ttaly | 2017 |
| 154 | Porcine circovirus 3 isolate 4289 | MF805722 | Italy | 2016 |
| 155 | Porcine circovirus 3 isolate 32941 | MF805721 | Italy | 2016 |
| 156 | Porcine circovirus 3 isolate 1621 | MF805719 | Italy | 2017 |
| 157 | PCV3/Thailand/PB01/17 | MG310152 | Thailand | 2017 |
| 158 | 2164 | KX458235 | USA | 2015 |
| 159 | 29160 | KT869077 | USA | 2015 |
| 160 | PCV3-US/MN2016 | KX898030 | USA | 2016 |
| 161 | PCV3-US/MO2015 | KX778720 | USA | 2015 |
| 162 | PCV3-US/SD2016 | KX966193 | USA | 2016 |
